# Engagement with the national electronic health records by people with Parkinson's disease

**DOI:** 10.3389/fnagi.2026.1817635

**Published:** 2026-06-04

**Authors:** Marijus Giraitis, Isabel Schwaninger, Ivana Paccoud, Messaline Fomo, Patricia Martins Conde, Evi Wollscheid-Lengeling, Bjoern Eskofier, John Torous, Rejko Krüger, Jochen Klucken

**Affiliations:** 1 Luxembourg Centre for Systems Biomedicine, University of Luxembourg, Esch-sur-Alzette, Luxembourg; 2 Centre Hospitalier de Luxembourg, Strassen, Luxembourg; 3 Luxembourg Institute of Health, Strassen, Luxembourg; 4 Observatoire National de la Santé, Strassen, Luxembourg; 5 Chair of AI-supported Therapy Decisions, LMU München, Munich, Germany; 6 Institute of AI in Medicine, LMU Hospital, Munich, Germany; 7 Institute of AI for Health, Helmholtz Zentrum München, Neuherberg, Germany; 8 Division of Digital Psychiatry, Department of Psychiatry, Beth Israel Deaconess Medical Center, Boston, MA, United States; 9 Harvard Medical School, Boston, MA, United States

**Keywords:** digital health technology, digital medicine, Electronic Health Record (EHR), Parkinson's disease, patient engagement

## Abstract

**Background:**

People with Parkinson's disease (PwPD) require coordinated, multidisciplinary care, which can be facilitated by Electronic Health Records (EHRs) enabling efficient information exchange and personalized decision-making. Understanding which patient-, healthcare provider- (HCP), and technology-related factors drive EHR engagement among older population with complex health needs is crucial for the successful adoption and advancement of digital transformation in medicine.

**Methods:**

Guided by the digital health empowerment framework, this cross-sectional study explored patient engagement with the national EHRs among 191 PwPD in Luxembourg, using validated instruments, including eHealth Literacy Questionnaire (eHLQ) and Health Information National Trends Survey (HINTS).

**Results:**

Findings from the descriptive and regression analysis showed that only 29.8% respondents engaged multiple times with their personal EHR in the previous year, 70.2% have not used it, including 40.8 % who have never access it since the launch of EHR system. Key factors associated with higher engagement with personal EHR included being born in Luxembourg, milder disease severity, and higher digital health literacy, as well as receiving support from HCPs to use personal EHR. Surprisingly, higher trust in HCPs and greater health literacy were linked to lower personal EHR usage.

**Conclusions:**

Personal EHR engagement among the vulnerable aging population is influenced by a complex interplay of patient, HCP, and technology-related factors, which must be addressed holistically to ensure inclusive usage and adherence to digital health tools.

## 1 Introduction

Parkinson's disease (PD) is the second most common neurodegenerative disorder, projected to affect over 12 million people worldwide within the next 15 years (Dorsey et al., 2018). The diverse motor and non-motor impairments require comprehensive, multidisciplinary care plans (Giladi et al., 2014), making efficient health information-sharing critical for timely decisions and high-quality care (Klucken et al., 2018). Accessible and structured health information could also reinforce patients' self-awareness, empower them to assume greater responsibility in shared decision-making and self-management (Hickmann et al., 2022; Grady and Gough, 2014).

With the growing innovation in the field of digital health technologies (DHT) (Boers et al., 2025), Electronic Health Records (EHR) have emerged as essential tools connecting healthcare providers (HCPs), patients, and digital services. EHRs are described as dynamic digital repositories that document patients' medical histories in real time and support up-to-date information exchange (ISO, 2019). EHRs have the potential to centralize information from multiple sources, providing users (patients and HCPs) with a single access point to personalized heath information. At present, these systems primarily contain standard clinical information from imaging and laboratory services, physician reports from hospitalizations and outpatient visits—which, in the context of PD, contain detailed information on the complexity of motor and non-motor symptoms, motor complications, treatment decisions, prescribed pharmacological and device-aided therapies amongst other health information (Ehrenstein et al., 2019; Soman et al., 2023; Academic Press, 2021). These reports are rich in clinical insight and indispensable for individual patient care. They are predominantly uploaded as heterogeneous free-text narratives, subject to physician's documentation habits, professional nomenclatures, and individual preferences, with the ultimate goal to systematically manage, support, coordinate care, and provide access to health data for secondary use for research (Klucken et al., 2018; Lee et al., 2020; Eskofier and Klucken, 2023; Raab et al., 2023).

Beyond organizational implications for patient-provider interactions, EHRs have attracted policymakers' attention at the pan-European level. The European Health Data Space (EHDS) regulation has recently entered into force, mandating all member states to implement national EHRs. It aims to empower citizens to generate, access, and share health data across borders (European Commission, 2025b), and in parallel provide “real-world” data for research and development (EPF, 2025). With the new interoperability standards, EHRs are further expected to integrate patient-generated health data from digital medical devices, such as wearable sensors and patient-facing mobile applications designed to inform, diagnose, manage, monitor or treat disease (Boers et al., 2025), allowing continuous, objectively monitored information to flow directly into personal EHRs and complement existing care data (Dinh-Le et al., 2019; Kawu et al., 2023).

In Luxembourg, a national EHR, *Dossier de Soins Partagé*, has been implemented since late 2019 by the national EHR agency (Agence eSanté Luxembourg). Since then, a personal EHR account is automatically created for all affiliated with the national social security system, including cross-border workers from the Greater Region [Lorraine (France), Rhineland-Palatinate and Saarland (Germany), and Wallonia (Belgium)]. Individuals retain the option to opt out or withdraw their personal EHR account at any time. The success and sustainability of this large-scale technology deployment relies on user acceptance—HCPs recognizing its clinical utility in decision-making process, and patients engaging with it as part of self-care routine (Sapienza et al., 2024; Nunes and Fitzpatrick, 2015).

Engagement with health technologies is defined as the extent to which individuals interact with and adhere to these technologies in ways that contribute to sustained behavior change and improved health outcomes (Zhang et al., 2025). Low user engagement remains a major challenge in digital healthcare transformation, delaying global adoption and compromising research validity due to insufficient data resulting from poor user adherence and retention (Goodday et al., 2024; Ng et al., 2019; Torous et al., 2018; Smith et al., 2025). A recent work has applied the capability framework and conceptualized how digital health technologies can empower patients to achieve valuable health outcomes by shifting attention from what technologies *can do*, to what patients are actually *able to achieve* with them. Crucial to this perspective is the role of conversion factors—defined as personal abilities, social, and environmental conditions—including technology and healthcare related factors, which can either facilitate or hinder the transition from mere use of technological resources into empowering capabilities, such as self-efficacy, self-awareness, self-management, and shared decision-making (Fomo et al., 2025). Notably, many reported user engagement barriers, such as physical and mental limitations, low digital and health literacy, trust, and privacy concerns, correspond closely with the conversion factors emphasized in the digital health empowerment framework, highlighting the need to identify and address these factors in order to design and deploy DHT that empower patients and sustain their engagement (Goodday et al., 2024; Torous et al., 2018; Fomo et al., 2025; Livieri et al., 2025).

Indeed, the effective engagement with personal EHRs requires both motivation and an adequate level of digital health literacy—ability to access, understand, and use health data obtained through DHT (Cheng et al., 2022b). This may be especially challenging for PwPD, who may require further technological and caregiver support, given their age, declining cognitive function and worsening functional impairments (EPF, 2025; Paccoud et al., 2024b; Esper et al., 2024). Encouragement and support by trusted HCPs may also serve as a critical enabler of health technology adoption. Their reassurance and guidance can help patients perceive personal EHRs as a safe extension of care rather than additional or burdensome tool (Ramachandran et al., 2023; Skeidsvoll Solvang et al., 2025).

To our knowledge, research on engagement with personal EHRs in the older populations is scarce in the current literature as these studies have mostly focused on evaluating personal EHR adoption among the general population (Agrawal et al., 2025; Xu et al., 2024; Paccoud et al., 2021), or leveraging real-word care information from EHRs for predicting PD diagnosis, monitoring treatment adherence or patient stratifications (Soman et al., 2023; Yi et al., 2024; Hill et al., 2023). While valuable, these efforts conceptualize EHRs predominantly as data repositories rather than patient-facing digital tools requiring active engagement. These approaches risk introducing systematic bias if the underlying data is not representative—particularly if individuals with a greater symptom burden, lower digital literacy, or limited access to digital tools are underrepresented among personal EHR users (de Graaf et al., 2017). Notably, studies that examine EHRs as digital interventions, focusing on how and why aging populations, like PwPD, engage with them are largely absent from the literature, highlighting a critical gap that EHRs are often treated as sources of health data rather than as tools with potential to deliver direct value to older people with complex care needs, who may be digitally excluded due to their vulnerability. Given the growing global interest in the EHRs, the complexity of PD offers a unique use-case for studying digital health engagement and adoption of national EHR systems among older people with a neurodegenerative disease. Failure to address these factors could risk widening existing health inequalities and deepen the digital divide, leaving out those individuals with the highest support needs (EPF, 2025; Esper et al., 2024; Paccoud et al., 2021). Therefore, the aim of this paper is to evaluate the usage of the national EHR among PwPD, describe their engagement characteristics, and by applying the digital empowerment framework, to identify patient-, HCP-, and technology-related conversion factors that determine patient engagement with personal EHR.

## 2 Methods

### 2.1 Data collection and sample

A cross-sectional survey was conducted by recruiting PwPD through the Luxembourg Parkinson's Study (NCER-PD) which hosts the largest cohort of PwPD in the country (Pavelka et al., 2023) as well as through patient associations in Luxembourg and the Greater Region. The study was advertised through email invitations, mailed flyers, and presentations at patient associations. Data collection took place between July 1^st^ 2024 and December 31^st^ 2024. Participants were eligible if they were above 18 years old, had a clinically confirmed diagnosis of Parkinson's disease, spoke English, French or German and provided informed consent.

After signing the informed consent form, participants were given an option to complete the survey either online or on paper in the three languages. Those opting for the online format received a personalized link via email to access a web-based survey page implemented within the REDCap platform (Vanderbilt University, RRID:SCR_003445) in survey mode, which is a secure electronic data capture software, commonly used in research environments (Oxford Academic, 2021). All study-related data were stored at the University of Luxembourg. The study received an Ethical approval from the Ethics Review Panel of the University of Luxembourg (ERP 25-010 MyPD) and the National Research Ethics Committee (N°202311/01 Version 4.0).

### 2.2 Instruments

#### 2.2.1 EHR usage and engagement

EHR usage and engagement characteristics were assessed using items from the Health Information National Trends Survey (HINTS) (Nelson et al., 2004), tailored for the study population and local digital services environment. The HINTS instrument evaluates participants' knowledge of, attitude toward, and use of health information technology. It includes categorical and ordinal variables as single-item questions to evaluate the practical aspects of engagement with digital health technology and health services. To measure the use and engagement with personal EHR we used the *frequency of EHR usage in the last 12 months* ranging from 0 to 10 times. Participants that have never accessed personal EHR and those who have accessed it in the past, but haven't used it in the last 12 months were classified as *non-users*, while those who have accessed personal EHR at least once in the last 12 months were classified as *users*.

#### 2.2.2 Patient-related factors

Socio-demographic and clinical variables were collected to characterize the sample and investigate patient-related conversion factors. The association of migration status was measured by country of birth variable which was grouped into 2 categories (“Born in Luxembourg”; “Not born in Luxembourg”). *Education* was grouped into 3 categories (“Low”; “Medium”; “High”) based on the ISCED (International Standard Classification of Education) 2011 classification (Eurostat, 2011). *Disease duration* was measured in years, grouped into 2 categories (“0–5 years”, and “≥6 years”), S*elf-reported disease severity* (Patient Global Impression of Severity: None; Mild; Moderate; Severe) (National Library of Medicine, 1976) was grouped into 2 categories (“None to Mild” and “Moderate to Severe”). *Income* was evaluated as perceived comfort living on present income, grouped into 3 categories (“Living comfortably on present income”, “Coping on present income”, “Finding it difficult or very difficult on present income”).

Digital Health Literacy was evaluated using five domains from the eHealth Literacy Questionnaire (eHLQ)—a validated multidimensional tool that has shown robust psychometric properties in populations with multiple chronic conditions (Kayser et al., 2018; Cheng et al., 2022a). eHLQ consists of 35 items grouped in seven domains (see Table 1 for eHLQ domains), where each item is scored on a 4-point Likert scale (1: Strongly disagree, 2: Disagree, 3: Agree, 4: Strongly agree). Individual scores for each domain were calculated by averaging the item scores within each domain with equal weighting, resulting in seven scores. Higher scores indicate greater confidence in using digital technologies and general health information, scores below 2.5 indicate low confidence. While the instrument hasn't been utilized in the Parkinson's context, it has been chosen due to its comprehensive and disease-agnostic properties in evaluating both individual- and technology-related factors that shape technology engagement.

**Table 1:** Sample characteristics grouped into patient-, healthcare professional-, and technology-related categories.

**Participant characteristics**	**Total sample (*n =* 191)**
**Patient-related factors**	
**Age, median (IQR)**	**67 (13)**
Age, *n* (%)	
< 60	38 (19.9%)
60–69	79 (41.4%)
70+	74 (38.7%)
Gender, *n* (%)	
Male	123 (64.6%)
Female	68 (35.6%)
Education, *n* (%)	
Low (ISCED 1–2)	33 (17.3%)
Medium (ISCED 3–4)	94 (49.2%)
High (ISCED 5–8)	64 (33.5%)
Country of birth, *n* (%)	
Luxembourg	62 (32.5%)
Not in Luxembourg	129 (67.5%)
Household income, *n* (%)	
Living comfortably on present income	100 (52.4%)
Coping on present income	76 (39.8%)
Finding it difficult or very difficult on present income	15 (7.8%)
Employment status, *n* (%)	
Actively working	29 (15.2%)
Not working	162 (84.8%)
Partnership status, *n* (%)	
Living in partnership	149 (78.1%)
Not living in partnership	42 (21.9%)
Household location, *n* (%)	
Urban	75 (39.3%)
Suburban	40 (20.9%)
Rural	76 (39.8%)
Disease duration, *n* (%)	
0–5 years	95 (49.7%)
≥6 years	96 (50.3%)
Self-reported disease severity, *n* (%)	
None to mild	78 (40.9%)
Moderate to severe	113 (59.2%)
Co-dependence on informal caregiver, *n* (%)	
Yes	71 (37.2%)
Digital health literacy (eHLQ)	
*Average score per domain (range 1–4), median (IQR)*	
1. Using technology to process health information	2.80 (0.6)
2. Understanding of health concepts and language	2.89 (0.6)
3. Ability to actively engage with digital services	3.00 (1.0)
4. Feel safe and in control	3.00 (0.6)
5. Motivated to engage with digital services	2.80 (0.8)
**Healthcare provider-related factors**	
Support from HCP for technology use, *n* (%)	
HCP offer support in EHR usage	40 (20.9%)
HCP encourage EHR usage	25 (13.1%)
Trust in HCPs, *n* (%)	
No to some	23 (12.0%)
Quite a lot	91 (47.6%)
A lot	77 (40.3%)
**Technology-related factors**	
*Average score per domain (range 1–4), median (IQR)*	
6. Access to digital services that work	2.33 (0.7)
7. Digital services that suit individual needs	2.50 (1.0)

SD, Standard deviation; ISCED, International Standard Classification of Education; IQR, Interquartile range; EHR, Electronic health record; HCP, Healthcare professional.

#### 2.2.3 Healthcare provider-related factors

Three variables were used to measure and explain the healthcare provider-related factors of personal EHR engagement: *Trust in HCPs, which* was grouped into 3 categories (“No to Some”; “Quite a lot”; “A lot”), *HCP offer support in EHR usage* (“Yes”, “No”), and *HCP encourage EHR usage* (“Yes”, “No”).

#### 2.2.4 Technology-related factors

Technology usability aspects were measured using two domains from the eHLQ questionnaire: *Access to digital services that work* and *Digital services that suit individual needs* (Kayser et al., 2018; Cheng et al., 2022a).

### 2.3 Data analysis

Data were analyzed using SPSS Statistics software (Version 25.0, IBM, RRID:SCR_002865). Descriptive statistics characterized the study population and EHR engagement. Group comparisons utilized chi-squared tests for categorical variables (with post-hoc comparisons and residual analysis where applicable), Kruskal-Wallis and Mann-Whitney U tests for non-normally distributed continuous (age and eHLQ domains score) and ordinal variables. Significant Kruskal-Wallis tests were followed by post hoc Dunn's tests. Bonferroni correction was applied to adjust for multiple comparisons (separately applied for 7 Digital Health Literacy subdomains, adjusted α < 0.007, and separately for 3 post hoc pairwise comparisons). Given that no substantial differences were observed between non-users and historic users in group comparisons, these two groups were merged into one global non-users group for regression analysis. Univariate and multivariate logistic regression models were applied to evaluate the relative associations of patient, HCP, and technology-related factors with personal EHR usage. A stepwise approach was used in multivariate models. All predictors within each domain were entered into three separate non-adjusted multivariate models, followed by a final adjusted model that included all variables across three domains. Age and gender were included as covariates in the final adjusted multivariate model. Odds ratios (OR) with 95% confidence interval (CI) estimated the association strength. Pseudo-*R*^2^ coefficients were used to assess and compare the relative performance of the regression models.

## 3 Results

### 3.1 Sample characteristics

Four hundred and fifty four PwPD were invited to complete the survey, 196 responses (43.2%) were collected over 6 months, 5 were incomplete, resulting in 191 surveys included in the analysis. The majority of participants were male (*n* = 123; 64.6%), 60 years old and above (*n* = 153; 80.1%) with medium (*n* = 94; 49.2%) and high education (*n* = 64; 33.5%), reporting high income (*n* = 100; 52.4%), moderate to severe disease severity (*n* = 113; 59.2%) and born outside of Luxembourg (*n* = 129; 67.5%). A summary of the study sample characteristics is presented in Table 1.

### 3.2 Engagement characteristics among EHR users

**Figure 1:**
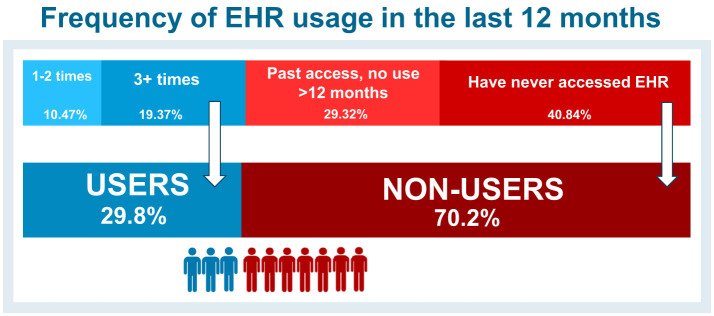
Frequency of EHR usage in the last 12 months among PwPD (*n* = 191). Users (*n* = 57; 29.8%) were defined as those with at least one use in the last 12 months, while non-users (*n* = 134; 70.2%) included those who have never accessed their personal EHR, or those who accessed their personal EHR in the past, but haven't used it the last 12 months.

**Figure 2:**
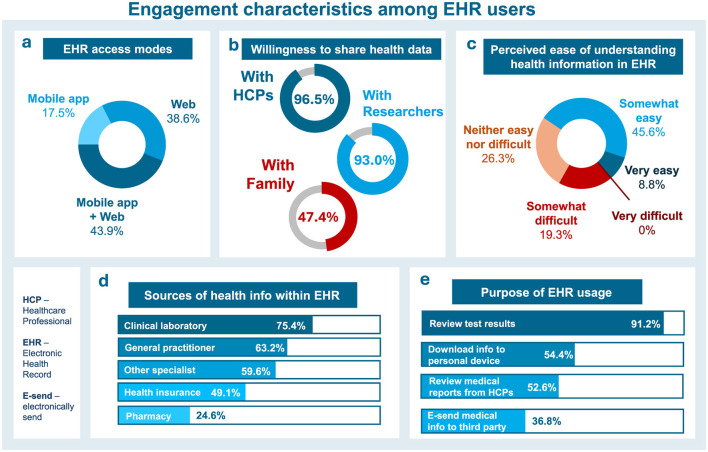
Engagement characteristics among EHR users (*n* = 57), including EHR access modes **(a)**; Willingness to share health data with HCPs, researchers, and family **(b)**; Perceived ease of understanding health information in EHR **(c)**; Sources of health information within EHR [**(d)**, multiple-choice question]; Purpose of EHR usage [**(e)**, multiple-choice question].

To understand how EHR users engage with their personal records, we examined usage frequency (Figure 1) and key engagement patterns (Figure 2). Less than half of participants (*n* = 78; 40.8%) have not accessed their personal EHR since the launch of the national EHR system (non-users). An additional, one-third of participants (*n* = 56; 29.3%) have accessed their personal EHR in the past, but haven't engaged with it in the past year (historic users). Taken together, this represent 70.2% of participants who can be classified overall as non-users. In contrast, about a third of participants (*n* = 57; 29.8%) have accessed their personal EHR at least once in the past year. Most of the EHR users have accessed it three or more times (*n* = 37; 19.4%) (current users).

We then described key engagement characteristics among current EHR users (Figure 2). From those who have used EHR (*n* = 57) at least once in the past year, more than half (*n* = 31; 54.4%) reported no difficulties in understanding the health information stored in personal EHR. Users commonly switched between access modes (*n* = 25, 43.9%) using both native mobile application and website interface. The most common reasons for using personal EHRs was to retrieve diagnostic test results (*n* = 52; 91.2%), followed by downloading health information to a personal device (*n* = 31; 54%) and reviewing medical reports from HCPs (*n* = 30; 53%). Even though sharing medical information with a third party (e.g. another HCP or family members) was the least common practice (*n* = 21; 36.8%), most users were willing to share their health information with HCPs (*n* = 55; 96.5%) and researchers (*n* = 53; 93.0%). However, less than half were accepting to share it with family (*n* = 27; 47.4%). A summary of the engagement characteristics is presented in Supplementary Table 5.

### 3.3 Conversion factors that determine engagement with personal EHR

To examine the differences in conversion factors for effective EHR usage, we compared current EHR users, historic users and non-users across a range of patient-, HCP-, and technology-related factor variables (Supplementary Table 1). No differences were observed between historic users and non-users, therefore, these two groups were merged into one global non-users group and compared to current users (Supplementary Table 2). Non-users were significantly more often born outside of Luxembourg (73.9%), reported moderate to severe disease severity (66.4%), and co-dependence on informal caregivers (42.5%), had lower confidence in digital health literacy (across five domains), reported not receiving HCP support (88.2%) neither HCP encouragement (95.5%) to use personal EHR, and generally reporting lower trust in HCPs (64.9% reported low-medium trust), compared to personal EHR users. Full results are presented in Supplementary Tables 1–3.

**Figure 3:**
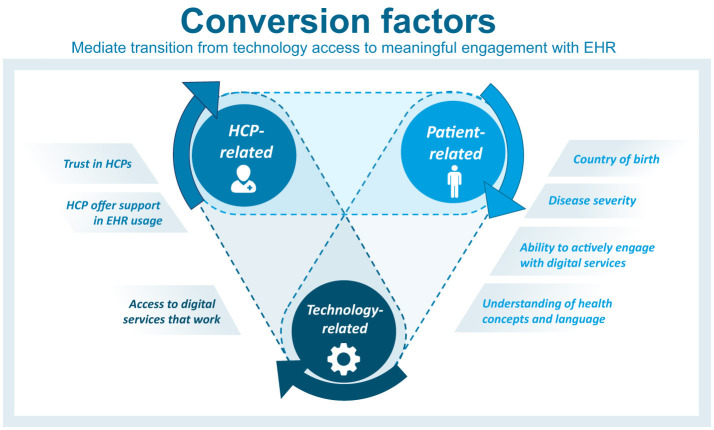
Identified patient-, healthcare provider (HCP)-, and technology-related conversion factors that determine engagement with the national EHRs among PwPD.

Next, to assess the magnitude and direction of the association between each conversion factor and personal EHR usage, multivariate logistic regression analyses were performed. Univariate analyses results are summarized in Supplementary Table 4. Multivariate analyses identified several, patient-, HCP-, and technology-related conversion factors associated with personal EHR usage (Table 2 and Figure 3). Individuals born in Luxembourg had higher odds of using personal EHR than those born in other countries (OR = 3.55, 95% CI = 1.39–9.08). Similarly, participants reporting milder self-perceived disease severity had higher odds of personal EHR usage (OR = 2.99, 95% CI = 1.23–7.26) then those with a higher disease severity. When looking into the associations with technology- and HCP-related factors, higher confidence in “*Ability to actively engage with digital services”* (OR = 4.39, 95% CI = 1.43–13.48), “*Access to digital services that works”* (OR = 4.67, 95% CI = 1.25–17.40), as well as *HCP offer support in EHR usage* (OR = 3.78, 95% CI = 1.32–10.73) were positively associated with personal EHR usage. However, in our study sample, participants with higher confidence in “*Understanding of health concepts and language”* (OR = 0.27, 95% CI = 0.09–0.84), and greater *trust in HCPs* (OR = 0.18, 95% CI = 0.05–0.66) were less likely to use EHR than their counterparts.

**Table 2:** Conversion factors associated with the higher odds of EHR usage.

Higher odds of EHR usage	Model 1 (patient-related factors)			Model 2 (HCP-related factors)			Model 3 (technology-related factors)			Model 4 (final adjusted model)		
	Odds ratio	95% CI	*P*-value	Odds ratio	95% CI	*P*-value	Odds ratio	95% CI	*P*-value	Odds ratio	95% CI	*P*-value
**Patient-related factors**												
Education, [ref. “High (ISCED 5–8)”]												
Low (ISCED 1–2)	0.44	0.13–1.47	0.18							0.33	0.08–1.34	0.12
Medium (ISCED 3–4)	0.65	0.29–1.45	0.29							0.46	0.18–1.19	0.11
Country of birth (ref. “Not in Luxembourg”)												
In Luxembourg	3.48	1.55–7.79	0.002^*^							3.55	1.39–9.08	0.008**
Household income, (ref. “Living comfortably on present income”)												
Coping on present income	1.70	0.76–3.79	0.20							1.79	0.69–4.66	0.23
Finding it difficult or very difficult on present income	3.85	0.92–16.07	0.06							4.79	0.79–29.27	0.09
Self-reported disease severity (ref. “Moderate” to “Severe”)												
None to mild	3.02	1.40–6.51	0.005^**^							2.99	1.23–7.26	0.02*
Co-dependence on informal caregiver (ref. “No”)												
Yes	0.69	0.30–1.58	0.38							0.88	0.33–2.33	0.79
Digital health literacy												
1. Using technology to process health information	1.23	0.43–3.49	0.69							0.88	0.26–3.02	0.84
2. Understanding of health concepts and language	0.38	0.15–0.99	0.049^*^							0.27	0.09–0.84	0.02*
3. Ability to actively engage with digital services	3.91	1.58–9.68	0.003^**^							4.39	1.43–13.48	0.01*
4. Feel safe and in control	1.03	0.49–2.18	0.93							0.68	0.27–1.71	0.42
5. Motivated to engage with digital services	1.54	0.54–4.36	0.42							0.73	0.18–2.93	0.65
**Healthcare provider-related factors**												
HCP offer support in EHR usage (ref. “No”)												
Yes				6.61	2.99–14.57	< 0.001^***^				3.78	1.32–10.73	0.01*
Trust in HCPs (ref. “No” to “Some”)												
Quite a lot				0.18	0.06–0.51	0.001^**^				0.12	0.04–0.44	0.001**
A lot				0.48	0.18–1.32	0.16				0.18	0.05–0.66	0.01*
**Technology-related factors**												
Access to digital services that work							2.88	1.18–7.02	0.02^*^	4.67	1.25–17.40	0.02*
Digital services that suit individual needs							1.41	0.63–3.12	0.40	1.27	0.38–4.19	0.70
Pseudo-*R^2^*	*0.316*			*0.248*			*0.149*			*0.479*		
Constant	*0.008*			*0.741*			*0.012*			*0.059*		
Goodness-of-fit test	*P = 0.88*			*P = 0.25*			*P = 0.44*			*P = 0.17*		

Multivariate logistic regression models. Models 1–3 are not adjusted for confounders. Model 4 adjusted for age and gender. Conversion factors were identified through multivariate logistic regression modeling; final model was adjusted for age and gender. Self-reported disease severity was evaluated with the Patient Global Impression of Severity instrument (PGI-S); Digital Health Literacy was measured with the eHealth Literacy Questionnaire (eHLQ); Healthcare provider-related factors were evaluated with the Health Information National Trends Survey (HINTS); Technology-related factors were measured with eHLQ questionnaire. EHR, Electronic health record; HCP, Healthcare professional; CI, Confidence interval. *P-value < 0.05; ^**^P-value < 0.01; ^***^P-value < 0.001.

Based on the results from our analysis, we propose an exploratory evaluation framework that could serve as a guidance when examining factors related to technology engagement among PwPD, and those affected by other chronic conditions (Figure 3).

## 4 Discussion

This cross-sectional study explored how PwPD, who often have complex care needs and may be at-risk of digital exclusion, engage with the national EHRs, taking Luxembourg's context as a case example. It allowed us to identify main conversion factors focusing on the key a) patient-, b) HCP-, and c) technology-related aspects. Our results showed that one-third of the respondents (aged 41–85) had accessed their personal EHRs, and one-fifth reported regular engagement in the last 12 months. This proportion is modestly higher than the 22% reported for the general Luxembourg population in 2024 by the European Observatory on Health Systems and Policies in its cross-country analysis of EHR uptake (European Commission, 2025c). This difference is expected as the Observatory data reflect a general population with lower healthcare needs compared to a clinical cohort of patients living with a progressive chronic condition, such as PD. Given a high cross-border patient mobility in Luxembourg and Greater Region, some patients may also be affiliated with neighboring social security systems, which may grant them access to national EHRs in Germany, *elecktronische Patientenakte (ePA)*, in France*, Dossier Médical Partagé*, or in Belgium*, Masanté*. Despite the existence of national EHRs throughout the Greater Region, their usage among PwPD in each country remains understudied, making comparisons with previous work limited.

However, other studies in the general population report high variability in personal EHR engagement, confirming many global deployment challenges. Five years after the national EHR rollout, usage among the general population reached 1.5% in France (Séroussi and Bouaud, 2017), 21% in Australia (Walsh et al., 2017), while in Estonia EHR usage reached 25% after 12 years of EHR rollout (Tiik and Ross, 2010). In contrast, studies in the United States of America (USA) report notably higher engagement ranging from 38% to 81.9%, that has been achieved over a 3–6 year period, depending on the EHR system and implementation context (HealthIT.gov, 2021; Shah and Fiala, 2025; Richwine, 2024). These variations likely reflect technical and strategic differences: EU countries often choose centralized national systems focused on long-term infrastructure, universal coverage, interoperability and cross-border standardization (Raab et al., 2023; European Commission, 2025a), whereas USA favors decentralized regional models, accelerated by federal incentives often deployed to specific diseases or population sub-groups (Burde, 2011; FDA, 2025). Despite rapid innovation, these large-scale deployments tend to prioritize technical progress over user acceptance and rarely achieve complete and sustainable uptake. Disparities in socio-economic, health status, and digital competences may further hinder adoption, yet remain under-studied in PD context. Their impact is further overlaid by patients' “speed of trust”—high trust in digital healthcare systems accelerates technology uptake and lowers resistance for changes, whereas low trust slows engagement and amplifies existing barriers (Covey and Merrill, 2006).

We found that among the users, majority of them (64.9%) accessed their personal EHRs at least three times in the past year, suggesting that once the initial connection barrier is crossed, users begin to integrate personal EHR into their routines. However, as chronic conditions progress, patients require more intensive care, making continuous EHR engagement more valuable, yet more challenging—reflected by non-users' more advanced disease and higher reliance on informal caregivers (42.5% of non-users require informal caregiver support) (Supplementary Table 2). Subgroup analysis between historic users (those who used EHR in the past, but disengaged) and non-users (those who have never accessed EHR) revealed no differences, suggesting that prior engagement effects may diminish over time with prolonged non-use. This may also reflect the heterogeneity within the historic user group, marked by varying levels and durations of prior engagement, resulting in profiles more closely resembling non-users than current users. These findings understore the importance of sustained engagement to support the adoption of EHR into self-management routines. Furthermore, milder disease severity was associated with roughly a threefold higher odds of personal EHR usage. This aligns with the digital health paradox: those with the greatest healthcare needs are the least likely to use technology (Paccoud et al., 2024a). While informal caregivers are considered a valuable resource to offset disease progression (Giladi et al., 2014), only 47% of EHR users were willing to share information with the family, underscoring complex patient–family dynamics, possibly reflecting elements of stigma, judgment or the desire to maintain autonomy and privacy (Schwaninger et al., 2021; Nurgalieva et al., 2020).

While older age, lower income and education have been associated with lower personal EHR usage (Cheng et al., 2022b; Paccoud et al., 2021; Hu et al., 2024), socio-economic disparities were less apparent in our sample. Most participants reported medium (49.2%) to high (33.5%) education and sufficient (39.8%) to high (52.4%) comfort with their present income, and no differences were observed between EHR users vs. non-users for education and income, which suggests comparable socio-economic status across the sample. Given that this study was conducted in the European healthcare context with universal health coverage, where EHR access is publicly available and free of charge, this might have reduced socio-economic barriers. In the absence of these obstacles, which are frequently observed in studies among racial and ethnic minorities in USA (Shah and Fiala, 2025), non-engagement with personal EHR in multi-cultural contexts like Luxembourg and the Greater Region, may also arise from cultural disparities, language barriers, different patient–provider communication norms, or low familiarity with local healthcare systems (Paccoud et al., 2021; Chiesa et al., 2019), especially in Luxembourg's context, given a substantial proportion of skilled and financially stable migrants settling in the country. This pattern was reflected in our findings, with lower EHR usage among migrants—people born outside Luxembourg accounted for 73.9% of non-users. However, the relatively higher educational status of our sample may limit the generalizability of these findings to more socio-economically disadvantaged populations, therefore these findings should be interpreted in the context of other high-income Western countries. Further research is needed to assess how EHR engagement varies across such groups and to estimate the magnitude of educational impact.

Contrary to the initial assumptions, higher health literacy, measured through confidence in the understanding of health concepts and language (eHLQ questionnaire), was negatively associated with personal EHR usage in our study (Table 2), whereas a recent Australian study conducted by Cheng et al. (2022b) identified it as a strong positive predictor of EHR usage. Our findings could suggest that highly literate individuals may navigate the healthcare system more efficiently and already achieve satisfactory care quality through direct patient-provider interactions, making supplementary tools like personal EHR less valuable. This could point to non-engagement as a deliberate choice—a pattern highlighted as a notable barrier in several studies among older adults (de Graaf et al., 2017; Bieg et al., 2022). However, if the initial connection to personal EHR is achieved, users appear equally efficient in using it, with 55% of users in our sample reporting no difficulty to understand information in personal EHR (HINTS instrument). While these counterintuitive findings open new avenues for future research, they should be interpreted with caution given the observational uncontrolled design and relatively wider confidence intervals, and thus require further validation. Confidence in actively engaging with digital services (eHLQ questionnaire) further emerged as a strong predictor of personal EHR usage, in line with previous work describing digital skills as key to technology adoption (Cheng et al., 2022b; Esper et al., 2024). It confirms the critical role of digital training and support in enabling PwPD to use the technology independently or, when needed, with coordinated and disease-specific support adapted to their physical and cognitive limitations. To further improve accessibility, patient–public involvement (PPI) strategies are essential, engaging patients, informal caregivers, and HCPs in participatory co-design so that digital tools are from the start designed to accommodate a broad diversity of users. This is particularly relevant for widely accessible systems such as national EHRs, which serve users across the entire spectrum of health status—from the general population with occasional care needs to people living with longstanding and progressively debilitating conditions like PD. Equally, digital solutions must align with HCP and hospital workflows, as they are primarily responsible for populating patients' records with accurate care information (ResearchGate, 2024; Monje et al., 2023; Grosjean et al., 2022).

We observed that most users accessed their personal EHR independently −56.1% reported no support from HCPs, and 66.3% were not encouraged by HCPs to do so, suggesting a subgroup of early adopters driven by intrinsic interest. Nevertheless, HCPs support remained influential and was associated with fourfold higher odds of personal EHR usage. However, HCPs with limited technological experience may perceive digital tools as interfering with direct care delivery (Bassi et al., 2012), believe that patients prefer in-person interactions, worry about losing the collaborative spirit (Lordon et al., 2020), or have concerns about data privacy (Naegele et al., 2024). Interestingly, USA-based clinicians offer digital tools more frequently than their international counterparts and generally report higher confidence in their skills, more training received and stronger technical support, although insurance coverage issues remain a notable barrier. In contrast, HCPs outside the USA highlight their limited digital literacy and privacy concerns (Torous, 2025). Although, even if support is provided for patients, limited access to meaningful content could undermine users' engagement with personal EHRs. We observed that only 52.6% of users review consultation reports, which is likely due to their limited availability within personal EHRs to begin with. Depending on the level of system integration, some HCPs may have to manually upload consultation reports—a common situation in Luxembourg, where many HCPs operate in private practices. This time-consuming process for HCPs may hinder their consistent report-sharing and potentially discourage continued personal EHR use among the patients. In contrast, in Germany, HCPs are now legally required to upload their reports to the EHR since October 2025 (BMG, 2025), which is expected to strengthen collaboration between patients and HCPs. Access to comprehensive records can improve patients‘ sense of involvement and control over their care, as highlighted by the USA-based “OpenNotes” initiative, which granted patients full access to their health records (FDA, 2025; Delbanco et al., 2012). Empirical evidence showed that 99% of patients wanted “OpenNotes” to continue, and no HCPs opted out (Delbanco et al., 2012). When providers are both capable and willing to promote digital tools, achieving mutual trust may be key to translating HCP efforts into patient engagement.

Trust is a complex construct in digital health, described as a facilitator of technology uptake, emerging as a result of positive experiences, or diminishing when digital services fail to meet patient expectations (Ramachandran et al., 2023; Schwaninger et al., 2021; Hilty et al., 2021). Our findings revealed that higher trust in HCPs was unexpectedly associated with lower odds of personal EHR usage, but users reported higher trust in HCPs than non-users, suggesting some confounding effects. This could also indicate that trust may develop through personal EHR engagement rather than preceding it. Previous studies have shown that personal EHR usage can foster patients' trust in HCPs by improving transparency (O'Neill et al., 2019), obtaining a better overall picture of their health, gaining a feeling of being understood and by being valued by the provider (Pisciotta et al., 2019). Trust in HCPs is often linked to higher satisfaction with care, which may reduce patients' motivation to independently verify care information and consult additional resources like personal EHRs. Interestingly, European studies report stronger associations between trust and care satisfaction compared to North American studies (Birkhäuer et al., 2017). Notably, the USA ranks low for trust, but high for treatment satisfaction across 29 industrialized countries, whereas Switzerland, Denmark, and UK consistently rank high on both measures (Blendon et al., 2014). This divergence in the ranking of USA may reflect high healthcare costs, system fragmentation, or socio-economic inequalities—factors less apparent in more integrated European healthcare systems. Given the absence of a control group, the findings should be interpreted as exploratory and hypothesis-generating. Further research is needed to examine how trust in technology, the personal care team, and the broader healthcare system drive patient's engagement with digital medicine, both individually and as interdependent factors (Adjekum et al., 2018).

EHR non-users reported the lowest confidence in technology-related domains—access to *digital services that work* and access to *digital services that suit individual needs*, whereas they showed relatively higher confidence in digital health literacy domains (Supplementary Table 2), suggesting that technological barriers may limit engagement more than personal capabilities, such as motivation, digital skills or safety. It aligns with existing literature describing “defective technology” as a key barrier for adoption (Adjekum et al., 2018). Nevertheless, users in our sample showed flexibility and efficiently engaged with both access points—native mobile app and web-based interfaces, whereas patients in USA access personal EHRs more commonly via computers (HealthIT.gov, 2021). The ability to switch between interfaces is particularly favorable in the PD context, where older age and disease-related impairments could otherwise hinder engagement.

The rapid expansion of digital technology and growing public awareness suggest that personal EHR usage is likely to evolve, highlighting the need for future work. Several questions remain open, including the need of a more nuanced understanding of non-engagement and, importantly, the drivers of disengagement among the prior users. Longitudinal monitoring of individual engagement trajectories may help to identify the critical transition points at which users move from initial to sustained use, as well as the thresholds of engagement duration and frequency required to maintain continued EHR use. Incorporating controlled and comparative study designs is needed to better disentangle the individual influence of key determinants and to strengthen validity of the findings. Future research should explore how PD-related physical and cognitive limitations influence engagement across the disease stages, and whether longer disease duration promotes EHR engagement due to increasing healthcare needs or on the contrary, limits usage due to accumulating disease burden. Qualitative investigations into dynamic patient-provider relationships could inform tailored education campaigns to motivate both patients and HCPs to engage with personal EHRs collaboratively. Additionally, personal EHR usage metadata from the national registries could provide a more objective perspective into engagement trends across the general population and disease-specific subgroups.

## 5 Strengths and limitations

To our knowledge, this is the first study to explore EHR engagement patterns and associated determinants among PwPD. The study further contributes novel insights by applying a digital empowerment framework and identifying a comprehensive set of factors, which capture three fundamentally distinct domains: patient-, HCP- and technology-related determinants. This is notable as most of existing research has focused on the general population engagement with EHRs, often overlooking the specific needs, capabilities and barriers faced by older adults living with chronic, progressive conditions, or has primarily utilized EHR-derived data for predicting care outcomes rather than engagement analysis. Despite the differences in clinical manifestations, PD may serve as a relevant model to other older populations with neurodegenerative conditions due to its progressive, multisystem nature and increasing reliance on healthcare services over time, underscoring the importance of effective engagement with digital tools, as means of modernizing traditional care delivery models.

However, our work is not without limitations. This study has been conducted in a small-scale healthcare setting with a majority of individuals reporting medium to high education and sufficient financial comfort with their present income. This however, reflects Luxembourg's socio-economic structure, marked by highly educated population, ranking among Europe's leaders in higher education (alongside Ireland and Cyprus) (Eurostat, 2025), and in income levels (comparable to Ireland, Switzerland, Norway) (Eurostat, 2026). Recruiting from a well-established cohort with notable prior research experience may have favored individuals with higher socioeconomic status, limiting the external validity and generalizability of the results to more heterogeneous, resource-constrained populations. Furthermore, the observational and uncontrolled nature of the study limits causal interpretations. Therefore, the findings should be considered exploratory and indicative of potential patterns rather than presenting robust associations universally applicable across all populations. In addition, the wide confidence intervals of regression estimates are likely attributable to the modest sample size, which limited the precision of the findings, calling for more cautious generalizability and future research to validate the findings in larger samples. The primary aim of this study was to capture patient perspectives, therefore it relies on self-reported information which is generally prone to self-selection and recall bias. Complementary study designs such as including objective usage metadata or the input from HCPs could strengthen and enrich the findings. Finally, we did not inquire non-users about the reasons for non-engagement with personal EHRs, which could possibly be related to administrative barriers or intentional non-usage. There is a possibility that some study participants may not have been able to access personal EHR for reasons unrelated to the study, i.e., administrative circumstances such as non-affiliation with the national social security system, opting out of the personal EHR account creation, or non-awareness of its existence.

## 6 Conclusions

This study contributes to the limited literature and methodology on PwPD engagement with the personal EHR aimed at fostering self-agency and active participation in care. Using Luxembourg as a case study, we explored key patient-, HCP-, and technology-related conversion factors—such as socio-economic background, disease severity, digital and health literacy, trust in HCPs and their support—as important determinants shaping personal EHR usage within a small-scale healthcare setting. These findings can inform patient empowerment strategies lead by public health policymakers, support personal EHR deployment in other regions, and guide HCPs in promoting digital tools among aging populations with complex healthcare needs. Results further highlight the importance of HCPs supporting their patients in technology usage, providing them with opportunities to develop digital skills and co-designing technologies with end-users—particularly migrants and those with greater disease severity. While these findings offer general guidance on key factors for EHR implementation, they should be interpreted within the context of the observational, uncontrolled design with modest sample size. Further research in larger, more diverse PD populations is needed to validate these findings and develop accessible, user-centered EHR systems that demonstrate clinical utility and sustain user engagement. If these technology engagement barriers are not addressed, digital health initiatives risk widening existing health inequities and leaving behind those who may benefit the most from digital tools like personal EHRs.

## Data Availability

The datasets presented in this article are not readily available because of applicable data protection restrictions. The data that supports the findings of this study is available only upon reasonable request from the corresponding author. Requests to access the datasets should be directed to Jochen Klucken, jochen.klucken@uni.lu.
