# Supplementary material for: Engagement with the national electronic health records by people with Parkinson's disease
Source: Front Aging Neurosci. 2026 Jun 4;18:1817635. doi: 10.3389/fnagi.2026.1817635 (PMC13312789; doi:10.3389/fnagi.2026.1817635)
Supplement: Supplementary file 1 [file Data_Sheet_1.pdf]

Supplementary material

Engagement with the National Electronic Health Records by People with Parkinson's Disease

Marijus Giraitis[1,2,3], Isabel Schwaninger[1], Ivana Paccoud[1,4], Messaline Fomo[1], Patricia Martins Conde[1,2], Evi Wollscheid-Lengeling[1,2], Bjoern Eskofier [5,6,7], John Torous[8,9], Rejko Krüger[1,2,3], and Jochen Klucken[1,2]✉

[1] Luxembourg Centre for Systems Biomedicine (LCSB), University of Luxembourg, Esch-sur-Alzette, Luxembourg
[2] Centre Hospitalier de Luxembourg (CHL), Strassen, Luxembourg
[3] Luxembourg Institute of Health (LIH), Strassen, Luxembourg
[4] Observatoire National de la Santé, Strassen, Luxembourg
[5] Chair of AI-supported Therapy Decisions, LMU München, Munich, Germany
[6] Institute of AI in Medicine, LMU Hospital, Munich, Germany
[7] Institute of AI for Health, Helmholtz Zentrum München, Neuherberg, Germany
[8] Division of Digital Psychiatry, Department of Psychiatry, Beth Israel Deaconess Medical Center, Boston, MA, United States
[9] Harvard Medical School, Boston, MA, USA

✉ Corresponding author
Prof. Dr. med. Jochen Klucken
Email: jochen.klucken@uni.lu

Supplementary Table 1. Group comparisons between EHR non-users, historic users and current users

**Participant characteristics**	**Total sample** (N=191)	**Non-Users** (Never used) (N=78)	**Historic Users** (>12 months) (N=56)	**Current Users** (<12 months) (N=57)	**Between-groups comparison**		**Pairwise comparisons**					
							**Non-Users VS Historic Users**		**Non-Users VS Current Users**		**Historic Users vs Current Users**	
					Test statistic H(df) or χ^2(df)	P-value	Test statistic	P-value	Test statistic	P-value	Test statistic	P-value
**PATIENT-RELATED FACTORS**												
**Age,** median (IQR)	67 (13)	67 (13)	66 (13)	68 (15)	H(2)=.22	.90	NA	NA	NA	NA	NA	NA
**Age,** n (%)					H(2)=.29	.87	NA	NA	NA	NA	NA	NA
<60	38 (19.9%)	14 (17.9%)	9 (16.1%)	15 (26.3%)								
60 – 69	79 (41.4%)	35 (44.9%)	25 (44.6%)	19 (33.3%)								
70+	74 (38.7%)	29 (37.2%)	22 (39.3%)	23 (40.4%)								
**Gender,** n (%)					$\chi^2(2)=3.01$	.22	NA	NA	NA	NA	NA	NA
Male	123 (64.6%)	45 (57.7%)	37 (66.1%)	41 (71.9%)								
Female	68 (35.6%)	33 (42.3%)	19 (33.9%)	16 (28.1%)								
**Education,** n (%)					H(2)=5.80	.06	NA	NA	NA	NA	NA	NA
Low (ISCED 1-2)	33 (17.3%)	9 (11.5%)	18 (32.1%)	6 (10.5%)								
Medium (ISCED 3-4)	94 (49.2%)	43 (55.1%)	22 (39.3%)	29 (50.9%)								
High (ISCED 5-8)	64 (33.5%)	26 (33.3%)	16 (28.6%)	22 (38.6%)								
**Country of birth,** n (%)					$\chi^2(2)=8.50$	.01*	$\chi^2(2)=.30$	.58	$\chi^2(2)=7.76$	.005**	$\chi^2(2)=4.23$	.04
Luxembourg	62 (32.5%)	19 (24.4%)	16 (28.6%)	27 (47.4%)								
Not in Luxembourg	129 (67.5%)	59 (75.6%)	40 (71.4%)	30 (52.6%)								
Germany	82 (42.9%)	48 (61.5%)	25 (44.6%)	9 (15.8%)								
France	19 (9.9%)	7 (9.0%)	5 (8.9%)	7 (12.3%)								
Belgium	13 (6.8%)	1 (1.3%)	4 (7.1%)	8 (14.0%)								
Other	15 (7.9%)	3 (3.9%)	6 (10.8%)	6 (10.5%)								
**Household income,** n (%)					H(2)=.35	.84	NA	NA	NA	NA	NA	NA
Living comfortably on present income	100 (52.4%)	39 (50.0%)	32 (57.1%)	29 (50.9%)								
Coping on present income	76 (39.8%)	36 (46.2%)	18 (32.1%)	22 (38.6%)								

Finding it difficult or very difficult on present income	15 (7.8%)	3 (3.8%)	6 (10.7%)	6 (10.5%)								
**Employment status,** n (%)					$\chi^2(2)$=.54	.97	NA	NA	NA	NA	NA	NA
Actively working	29 (15.2%)	12 (15.4%)	8 (14.3%)	9 (15.8%)								
Not working	162 (84.8%)	66 (84.6%)	48 (85.7%)	48 (84.2%)								
**Partnership status,** n (%)					$\chi^2(2)$=4.17	.12	NA	NA	NA	NA	NA	NA
Living in partnership	149 (78.1%)	58 (74.4%)	49 (87.5%)	42 (73.7%)								
Not living in partnership	42 (21.9%)	20 (25.6%)	7 (12.5%)	15 (26.3%)								
**Household location,** n (%)					$\chi^2(2)$=9.4	.05	NA	NA	NA	NA	NA	NA
Urban	75 (39.3%)	38 (48.7%)	18 (32.1%)	19 (33.3%)								
Suburban	40 (20.9%)	11 (14.1%)	18 (32.1%)	11 (19.3%)								
Rural	76 (39.8%)	29 (37.2%)	20 (35.7%)	27 (47.4%)								
**Disease duration,** n (%)					$\chi^2(2)$=1.50	.47	NA	NA	NA	NA	NA	NA
0-5 years	95 (49.7%)	41 (52.6%)	24 (42.9%)	30 (52.6%)								
≥6 years	96 (50.3%)	37 (47.4%)	32 (57.1%)	27 (47.4%)								
**Self-reported disease severity**, n (%)					$\chi^2(2)$=9.87	.007*	$\chi^2(2)$=.09	.77	$\chi^2(2)$=7.23	.007**	$\chi^2(2)$=7.57	.006**
None to Mild	78 (40.9%)	27 (34.6%)	18 (32.1%)	33 (57.9%)								
Moderate to Severe	113 (59.2%)	51 (65.4%)	38 (67.9%)	24 (42.1%)								
**Co-dependence on informal caregiver,** n (%)					$\chi^2(2)$=5.54	.06	NA	NA	NA	NA	NA	NA
Yes	71 (37.2%)	33 (42.3%)	24 (42.9%)	14 (24.6%)								
**Digital health literacy** Average score per domain (range 1-4), median (IQR)												
1. Using technology to process health information	2.80 (0.6)	2.79 (0.6)	2.60 (0.8)	3.00 (0.9)	H(2)=11.29	.004**	Z=-3.93	.68	Z=-30.59	.001**	Z=-26.66	.01**
2. Understanding of health concepts and language	2.89 (0.6)	3.00 (0.6)	2.80 (0.4)	3.00 (0.8)	H(2)=4.48	.11	NA	NA	NA	NA	NA	NA

3. Ability to actively engage with digital services	3.00 (1.0)	2.80 (1.0)	2.80 (1.2)	3.20 (0.9)	H(2)=16.57	<.001**	Z=-4.84	.62	Z=-37.17	<.001**	Z=-32.33	.002**
4. Feel safe and in control	3.00 (0.6)	2.80 (0.4)	3.00 (0.6)	3.00 (0.7)	H(2)=7.31	.03	NA	NA	NA	NA	NA	NA
5. Motivated to engage with digital services	2.80 (0.8)	2.80 (0.6)	2.80 (0.8)	3.00 (0.8)	H(2)=9.59	.008	Z=-13.11	.17	Z=-29.62	.002	Z=-16.51	.11
**HEALTHCARE PROVIDER-RELATED FACTORS**												
**Support from HCP for technology use,** n (%)												
HCP offer support in EHR usage	40 (20.9%)	5 (6.4%)	10 (17.9%)	25 (43.9%)	$\chi^2(2)$=28.35	<.001**	$\chi^2(2)$=4.30	.04	$\chi^2(2)$=26.72	<.001**	$\chi^2(2)$=8.93	.003**
HCP encourage EHR usage	25 (13.1%)	1 (1.3%)	5 (8.9%)	19 (33.3%)	$\chi^2(2)$=30.95	<.001**	$\chi^2(2)$=4.46	.04	$\chi^2(2)$=26.81	<.001**	$\chi^2(2)$=10.06	.002**
**Trust in HCPs,** n (%)					H(2)=5.44	.07	NA	NA	NA	NA	NA	NA
No to Some	23 (12.0%)	5 (6.4%)	7 (12.5%)	11 (19.3%)								
Quite a lot	91 (47.6%)	40 (51.3%)	35 (62.5%)	16 (28.1%)								
A lot	77 (40.3%)	33 (42.3%)	14 (25.0%)	30 (52.6%)								
**TECHNOLOGY-RELATED FACTORS**												
**Digital health literacy** Average score per domain (range 1-4), median (IQR)												
6. Access to digital services that work	2.33 (0.7)	2.33 (0.7)	2.50 (0.6)	2.83 (0.8)	H(2)=18.09	<.001**	Z=-12.47	.20	Z=-40.46	<.001**	Z=-27.99	.007**
7. Digital services that suit individual needs	2.50 (1.0)	2.25 (0.8)	2.50 (1.0)	3.00 (0.9)	H(2)=18.36	<.001**	Z=-13.73	.15	Z=-40.68	<.001**	Z=-26.96	.009**

ISCED – International Standard Classification of Education, HCP – Healthcare provider, PD – Parkinson's disease, IQR – Interquartile range, EHR – Electronic Health Record, df – degrees of freedom. Kruskal-Wallis test was applied for ordinal and continuous non-normally distributed variables, and, where applicable, followed by post-hoc Dunn's test for 3 pairwise comparisons. Global Chi-

squared test was applied for categorical variables, and where applicable, followed by 3 pairwise comparisons. *- Statistically significant at P-value <.05, **- Statistically significant after Bonferroni correction for multiple comparisons in respective categories (for Digital Health Literacy subdomains adjusted α levels=.007, for 3 post-hoc pairwise comparisons adjusted α levels = .017).

Supplementary Table 2. Group comparison between EHR users and non-users

Participant characteristics	Total sample (N=191)	Non-Users (N=134)	Users (N=57)	Between-group comparison	
				Test statistic U or χ²(df)	P-value
**PATIENT-RELATED FACTORS**					
**Age,** median (IQR)	67 (13)	66 (13)	68 (15)	U=3799.50	.96
**Age,** n (%)				$\chi^2(2)$=3.00	.22
<60	38 (19.9%)	23 (17.2%)	15 (26.3%)		
60 – 69	79 (41.4%)	60 (44.8%)	19 (33.3%)		
70+	74 (38.7%)	51 (38.1%)	23 (40.4%)		
**Gender,** n (%)				$\chi^2(1)$=2.01	.16
Male	123 (64.6%)	82 (61.2%)	41 (71.9%)		
Female	68 (35.6%)	52 (38.8%)	16 (28.1%)		
**Education**, n (%)				$\chi^2(2)$=2.82	.25
Low (ISCED 1-2)	33 (17.3%)	27 (20.1%)	6 (10.5%)		
Medium (ISCED 3-4)	94 (49.2%)	65 (48.5%)	29 (50.9%)		
High (ISCED 5-8)	64 (33.5%)	42 (31.3%)	22 (38.6%)		
**Country of birth,** n (%)				$\chi^2(1)$=8.24	.004*
Luxembourg	62 (32.5%)	35 (26.1%)	27 (47.4%)		
Not in Luxembourg	129 (67.5%)	99 (73.9%)	30 (52.6%)		
Germany	82 (42.9%)	73 (54.5%)	9 (15.8%)		
France	19 (9.9%)	12 (9.0%)	7 (12.3%)		
Belgium	13 (6.8%)	5 (3.7%)	8 (14.0%)		
Other	15 (7.9%)	9 (6.7%)	6 (10.5%)		
**Household income,** n (%)				$\chi^2(2)$=0.80	.67
Living comfortably on present income	100 (52.4%)	71 (53%)	29 (50.9%)		
Coping on present income	76 (39.8%)	54 (40.3%)	22 (38.6%)		
Finding it difficult or very difficult on present income	15 (7.8%)	9 (6.7%)	6 (10.6%)		
**Employment status,** n (%)				$\chi^2(1)$=0.23	.88
Actively working	29 (15.2%)	20 (14.9%)	9 (15.8%)		
Not working	162 (84.8%)	114 (85%)	48 (84.2%)		
**Partnership status,** n (%)				$\chi^2(1)$=0.89	.35
Living in partnership	149 (78.1%)	107(79.9%)	42 (73.7%)		
Not living in partnership	42 (21.9%)	27 (20.1%)	15 (26.3%)		
**Household location,** n (%)				$\chi^2(1)$=2.00	.37
Urban	75 (39.3%)	56 (41.8%)	19 (33.3%)		
Suburban	40 (20.9%)	29 (21.6%)	11 (19.3%)		
Rural	76 (39.8%)	49 (36.6%)	27 (47.4%)		

**Disease duration,** n (%)				$\chi^2(1)=0.27$	.60
0-5 years	95 (49.7%)	65 (48.5%)	30 (52.6%)		
≥6 years	96 (50.3%)	69 (51.5%)	27 (47.4%)		
**Self-reported disease severity**, n (%)				$\chi^2(1)=9.78$	.002*
None to Mild	78 (40.9%)	45 (33.6%)	33 (57.9%)		
Moderate to Severe	113 (59.2%)	89 (66.4%)	24 (42.1%)		
**Co-dependence on informal caregiver,** n (%)				$\chi^2(1)=5.53$	.019*
Yes	71 (37.2%)	57 (42.5%)	14 (24.6%)		
**Digital health literacy**<br>*Average score per domain (range 1-4), median (IQR)*					
1. Using technology to process health information	2.80 (0.6)	2.60 (0.6)	3.00 (0.9)	U=2661.50	<.001**
2. Understanding of health concepts and language	2.89 (0.6)	2.80 (0.6)	3.00 (0.8)	U=3320.50	.15
3. Ability to actively engage with digital services	3.00 (1.0)	2.80 (1.0)	3.20 (0.9)	U=2413.50	<.001**
4. Feel safe and in control	3.00 (0.6)	2.80 (0.6)	3.00 (0.7)	U=3035.50	.02
5. Motivated to engage with digital services	2.80 (0.8)	2.80 (0.6)	3.00 (0.8)	U=2853.50	.005**
**HEALTHCARE PROVIDER-RELATED FACTORS**					
**Support from HCP for technology use,** n (%)					
HCP offer support in EHR usage	40 (20.9%)	15 (11.2%)	25 (43.9%)	$\chi^2(1)=25.77$	<.001*
HCP encourage EHR usage	25 (13.1%)	6 (4.5%)	19 (33.3%)	$\chi^2(1)=29.27$	<.001*
**Trust in HCPs,** n (%)				$\chi^2(2)=13.14$	.001**
No to Some	23 (12.0%)	12 (9.0%)	11 (19.3%)		
Quite a lot	91 (47.6%)	75 (56.0%)	16 (28.1%)		
A lot	77 (40.3%)	47 (35.1%)	30 (52.6%)		
**TECHNOLOGY-RELATED FACTORS**					
**Digital health literacy**<br>Average score per domain (range 1-4), median (IQR)					
6. Access to digital services that work	2.33 (0.7)	2.33 (0.7)	2.83 (0.8)	U=2409.50	<.001**

7. Digital services that suit individual needs	2.50 (1.0)	2.25 (0.8)	3.00 (0.9)	U=2421.50	<.001**

ISCED – International Standard Classification of Education, HCP – Healthcare provider, PD – Parkinson's disease, IQR – Interquartile range, EHR – Electronic Health Record, df – degrees of freedom. *Statistically significant at P-value <.05, ** – Statistically significant after Bonferroni correction for multiple comparisons (for digital health literacy adjusted α levels = .0071; for trust in HCPs variable adjusted α levels = .017).

Supplementary Table 3. Post-hoc analyses of adjusted standardized residuals for "*Trust in HCPs*" and *EHR usage*

"Trust in HCPs" **variable categories**	**Test statistic**	**P-value**	**Z-score**	
			**Non-Users** (n=134)	**Users** (n=57)
No to Some	$\chi^2(2)=13.14$	.001**	-2.0	2.0
Quite a lot			3.5**	-3.5**
A lot			-2.3	2.3

HCP – Healthcare provider, EHR – Electronic Health Record. ** – Statistically significant after Bonferroni correction for multiple comparisons (adjusted α level = .017)

Supplementary Table 4. Factors associated with the higher odds of EHR usage. Individual factors were identified using univariate logistic regression, not adjusted for confounders.

Likelihood of EHR usage	Odds ratio	95% CI	P-value
**PATIENT-RELATED FACTORS**			
**Age** (ref. "70+")			
<60	1.45	0.64- 3.27	.38
60 – 69	0.70	0.34- 1.43	.33
**Gender** (ref. "Male")			
Female	0.62	0.31- 1.21	.16
**Education** (ref. "High (ISCED 5-8)")			
Low (ISCED 1-2)	0.42	0.15- 1.18	.10
Medium (ISCED 3-4)	0.85	0.43- 1.68	.64
**Country of birth** (ref. "Not in Luxembourg")			
In Luxembourg	2.55	1.33 – 4.86	.005*
**Household income** (ref. "Living comfortably on present income")			
Coping on present income	1.00	0.51- 1.93	.99
Finding it difficult or very difficult on present income	1.63	0.53- 5.00	.39
**Employment status** (ref. "Not working")			
Actively working	1.07	0.45 – 2.52	.88
**Partnership status** (ref. "Living in partnership")			
Not living in partnership	1.42	0.69 – 2.92	.35
**Household location** (ref. "Urban")			
Suburban	1.12	0.47 – 2.66	.80
Rural	1.62	0.81 – 3.27	.18
**Disease duration** (ref. "0-5 years")			
≥6 years	1.18	0.63 – 2.19	.60
**Self-reported disease severity** (ref. "Moderate" to "Severe")			
None to Mild	2.72	1.44 – 5.14	.002*
**Co-dependence on informal caregiver** (ref. "No")			
Yes	0.44	0.22 – 0.88	.02*
**Digital Health Literacy**			
1. Using technology to process health information	2.30	1.33 – 3.97	.003*
2. Understanding of health concepts and language	1.51	0.83 – 2.73	.18
3. Ability to actively engage with digital services	2.84	1.67 – 4.82	<.001*
4. Feel safe and in control	1.69	0.95 – 3.00	.07
5. Motivated to engage with digital services	2.29	1.28 – 4.10	.005*
**HEALTHCARE PROVIDER-RELATED FACTORS**			
**HCP offer support in EHR usage** (ref. "No")			
Yes	6.20	2.93 – 13.12	<.001*
**HCP encourage EHR usage** (ref. "No")			
Yes	10.67	3.98 – 28.61	<.001*

**Trust in HCPs** (ref. "No" to "Some")			
Quite a lot	0.23	0.87 – 0.62	.004*
A lot	0.70	0.27 – 1.78	.45
**TECHNOLOGY-RELATED FACTORS**			
**Digital Health Literacy**			
6. Access to digital services that work	3.81	2.04 – 7.10	<.001*
7. Digital services that suit individual needs	2.82	1.63 – 4.89	<.001*

HCP – Healthcare provider, EHR – Electronic Health Record, CI – Confidence intervals, * – Statistically significant at $\alpha < .05$.

Supplementary Table 5. Descriptive statistics of EHR engagement characteristics among EHR users

**Engagement characteristics**	**EHR users** (n=57)
**Access mode,** n (%)	
Mobile application	10 (17.5%)
Internet Website	22 (38.6%)
Both access modes	25 (43.9%)
**Purpose of EHR usage,** n (%)	
Review test results	52 (91.2%)
Review medical reports from HCP	30 (52.6%)
Download information to personal device	31 (54.4%)
Electronically send medical information to a third party	21 (36.8%)
**Sources of health information within EHR,** n (%)	
Clinical laboratory	43 (75.4%)
General practitioner	36 (63.2%)
Other medical specialist	34 (59.6%)
Health insurance	28 (49.1%)
Pharmacy	14 (24.6%)
**Perceived ease of understanding health information in EHR,** n (%)	
Very easy	5 (8.8%)
Somewhat easy	26 (45.6%)
Neither easy nor difficult	15 (26.3%)
Somewhat difficult	11 (19.3%)
Very difficult	0 (0%)
**Willingness to share health information,** n (%)	
With HCP	55 (96.5%)
With researchers	53 (93.0%)
With family	27 (47.4%)

EHR – Electronic health record, HCP – Healthcare provider
